# An analysis of the driving forces behind the Yangtze River Delta (YRD) region’s high-quality growth in the digital creative industries

**DOI:** 10.1371/journal.pone.0313647

**Published:** 2024-11-22

**Authors:** Xin Wang, Shan Sun, Yanlong Guo, Tieqiao Xiao

**Affiliations:** 1 Anhui Cultural Tourism Innovation Development Research Institute, Anhui Jianzhu University, Hefei, China; 2 School of Arts, Anhui Jianzhu University, Hefei, China; 3 Social Innovation Design Research Centre, Anhui University, Hefei, China; 4 School of Architecture and Planning, Anhui Jianzhu University, Hefei, China; Australian Catholic University, AUSTRALIA

## Abstract

The digital creative sector has grown enormously on a global scale as a result of the digital age’s fast progress. The development and transformation of the regional financial system in China is largely due to the efforts of the digital innovation industry. Hence, a comprehensive examination of the catalysts at the back of its premium boom is fundamental. The Yangtze River Delta (Abbreviation YRD) area is a powerful group of Chinese cities, well-known for its economic impact, the basis of its urbanization, and its total potential. It also offers some advantages related to the digital creative sector. The YRD area’s digital creative industries are growing, and this research looks at what’s behind that development. Through the compilation and analysis of surface-level data, the research focused on identifying the key drivers influencing the industry’s high-quality advancement. This is achieved by assessing the digital creative industries of the YRD and determining the elements that facilitate their continued growth and superior development using ArcMap10.8 and the entropy-weight-TOPSIS approach. This approach supports the assessment and ranking of the variables impacting the expansion of the digital creative industries in the YRD region. The findings demonstrate that: 1) The YRD region’s performance in developing a high-quality digital creative industry is improving between 2017 and 2022; 2) According to an assessment of growth in this sector, Jiangsu and Zhejiang provinces have excelled, while Shanghai and Anhui provinces have lagged in developing digital creative industries over the past six years; 3) Anhui and Zhejiang provinces had the greatest outcomes, according to an annual study of the YRD digital creative industries conducted between 2017 and 2022. The digital creative sector in Zhejiang Province has performed well, showing a consistent growing trend; in contrast, Shanghai City’s industry has seen a decrease; while Anhui and Jiangsu Provinces have shown generally stable, slightly increasing tendencies; 4) In the weighting analysis of the indicators, the number of strategic emerging industry project transactions (C10), the total income of radio and television business units (C13), the number of ordinary undergraduate and specialist graduates of ordinary schools (C18), the area of land for public facilities (C24), and the regional gross domestic product (C12) are five indicators of significant impact on the development of the degree of influence.

## Introduction

The digital creative sector has shown significant development potential as a key engine for both cultural innovation and economic progress in light of the rapidly expanding global digital economy. Because of its exceptional technical innovation skills, rich cultural resources, and robust economic power, China’s YRD area has emerged as a key hub for the growth of digital creative industries. The digital economy in the YRD has grown to be larger than 30% of the national economy, with the digital creative sector playing a significant role and showing high development momentum, according to the China Digital Economy Development Report 2024 [1]. This region is becoming an important force in leading the country’s economic transformation and innovation with strong support from national policies.

Due to the impact of the New Crown epidemic, consumption in the offline economy has decreased, while the widespread popularization of the Internet and the development of digital media platforms have led to the gradual growth of the digital creative industries, which have played an important role in promoting economic recovery and job creation [2]. Global economic data also demonstrate the importance of the creative and digital cultural sectors for global public management as well as their considerable economic effect [3, 4]. The Chinese government has progressively put in place a number of measures to encourage the growth of these businesses because of the high technical content and high value-added features of the creative industries. Major developed cities like Beijing, Shanghai, Guangzhou, Shenzhen, and Hangzhou have seen extraordinary growth in their creative industries, leading to the emergence of several creative clusters and businesses.

The digital creative industry is essentially an industry that uses digital technology and creative thinking to create, develop and distribute content and products [5]. In his book Digital Survival, Nicholas Negroponte describes the intersection of the computer, publishing and printing, broadcasting and film industries in three overlapping circles, and this intersection is none other than the digital creative industry, which is one of the fastest-growing and most innovative industries [6]. In contrast, the UK is leading the way in digital creativity with a much deeper cultural base, focusing primarily on the expansion of the digital content business and supporting the development of Korea and Japan in the convergence of digital and culture. The copyright business in the US, on the other hand, is the most important part of this and is at the centre of the synergistic advancement of digital content and technology [7].

Present-day academics mostly concentrate on strategies for advancing the growth of digital creative industries. Research has indicated that the inventive growth of the digital creative industries is significantly influenced by a number of factors, including government subsidies, R&D expenditure, and the innovation environment [8, 9]. Qi [10] points out that competition in technological development has become an important way to enhance the competitiveness of creative industries. Chinese academic Zang Zhipeng concurs that technology innovation fosters the growth of digital creative industries by offering resources and techniques that are more sophisticated as well as scientific and technological assistance [2]. Sun Shouqian proposes that content creation, cultural innovation, and copyright exploitation are the three key routes for the growth of digital creative industries based on the current state of development of these industries in China [11]. Hosseini, E [12] adds that by prioritising innovation, design and creativity, creative industries can enhance their competitiveness and thus take a better position in the global value chain.

From the traditional location theory and the history of industrial development, the regional socio-economic level is the key foundation for the flourishing development of digital creative industries [4, 13]. Mao and Qiu’s research shows that the regional socio-economic foundation influences the development of digital creative industries [14, 15]. Therefore, several factors, such as industrial structure, social environment and local economic conditions, work together to influence the development of digital creative industries [16, 17]. Market demand is the core driving force to promote the continuous progress of the industry. In order to satisfy the preferences and expectations of users and consumers, the market demand of digital creative industries is constantly changing and expanding, so it is necessary to formulate an overall development strategy in order to clarify the future direction of the industry [11].

When analysing the factors influencing the development of creative industries, Hosseini, E [12] and Crompton [18] state that talent is one of the most important variables. Creative talent is the driving force of the digital creative industry, and the talent pool is able to allocate resources efficiently and is responsible for developing and training future talent to support the industry in achieving its goals [18]. Various social forces should work together to support the growth and sustainability of the creative industries and integrate these efforts with ecological sustainability [19, 20]. As can be seen, many scholars’ studies not only focus on the impact of single factors on industrial innovation, but also emphasise the role of the integration of creative technologies, policies, markets, talents and local resources in promoting new digital creative industries [21, 22]. As there are differences in environment, policy, investment, technology and talent in different regions, leading to the uneven development of regional digital industries, it is imperative to study the factors affecting the high-quality development of digital creative industries.

Though the significance of the digital creative sector has been generally acknowledged, the majority of studies that have been done thus far concentrate on specific drivers, such as the role that technology innovation or policy support play, and do not provide a thorough examination of how these factors interact. Niu Junjun et al. point out that differences in regional policies and market environments are key factors affecting the development of the industry, but there is a lack of systematic research on the interactions of these factors across regions [23]. Liliya Satalkina and Gerald Steiner’s research further suggests that innovation ecosystems play a crucial role in the digital transformation of the cultural and creative industries in Europe, yet research in this area in China still lacks a comprehensive analysis of the interaction of multiple drivers. role in the digital transformation of cultural and creative industries in Europe, however, research in this area in China is still insufficient [24]. In addition, the YRD, as the core region of digital creative industries, has significant differences in its internal distribution and development level, and how these differences affect the development of regional industries has not been fully studied.

In light of the significant role that digital creative industries play in regional economic transformation, understanding the key drivers behind their growth is critical. While much research has focused on specific factors such as technological innovation or policy support, there is a lack of comprehensive analysis that examines how these factors interact in the context of the YRD region. Furthermore, given the YRD’s strategic importance to China’s digital economy, it is crucial to explore how these internal regional disparities influence the overall development of the digital creative industries. This study aims to fill this gap by systematically analyzing the main driving forces behind the high-quality development of digital creative industries in the YRD, providing valuable insights for regional policymakers and offering a foundation for future industrial development strategies.

The entropy-weight-TOPSIS approach was selected to systematically investigate these key drivers, allowing for a comprehensive evaluation of the effects of market, policy, technological innovation, and human resources. This method integrates the synergistic effects of these multiple drivers into a single analytical framework, contributing to theoretical research and serving as a resource for industrial policy-making in other regions. The entropy-weight-TOPSIS method has been proven effective in other fields, such as Raman Kumar et al.’s study on industrial optimization, which demonstrates its wide applicability in multi-criteria decision-making [25]. Similarly, Teri Silvio’s research on the creative industries in Korea and Japan highlights the importance of combining governmental support with market mechanisms—insights that resonate with this study’s synergy analysis [26].

As the center of China’s digital economy, the YRD region possesses significant natural resources and talent advantages, making it crucial for the growth of digital creative industries [27]. However, despite its recent growth, the development of digital creative industries in the region has not yet matched its economic potential [1]. Thus, it is essential to explore the factors driving the high-quality growth of the YRD’s digital creative industries. This study aims to assess these internal drivers and provide insights for future development strategies to encourage sustainable growth in the YRD’s digital creative industries.

## Materials and methods

### The scope of the area of research

**Fig 1:**
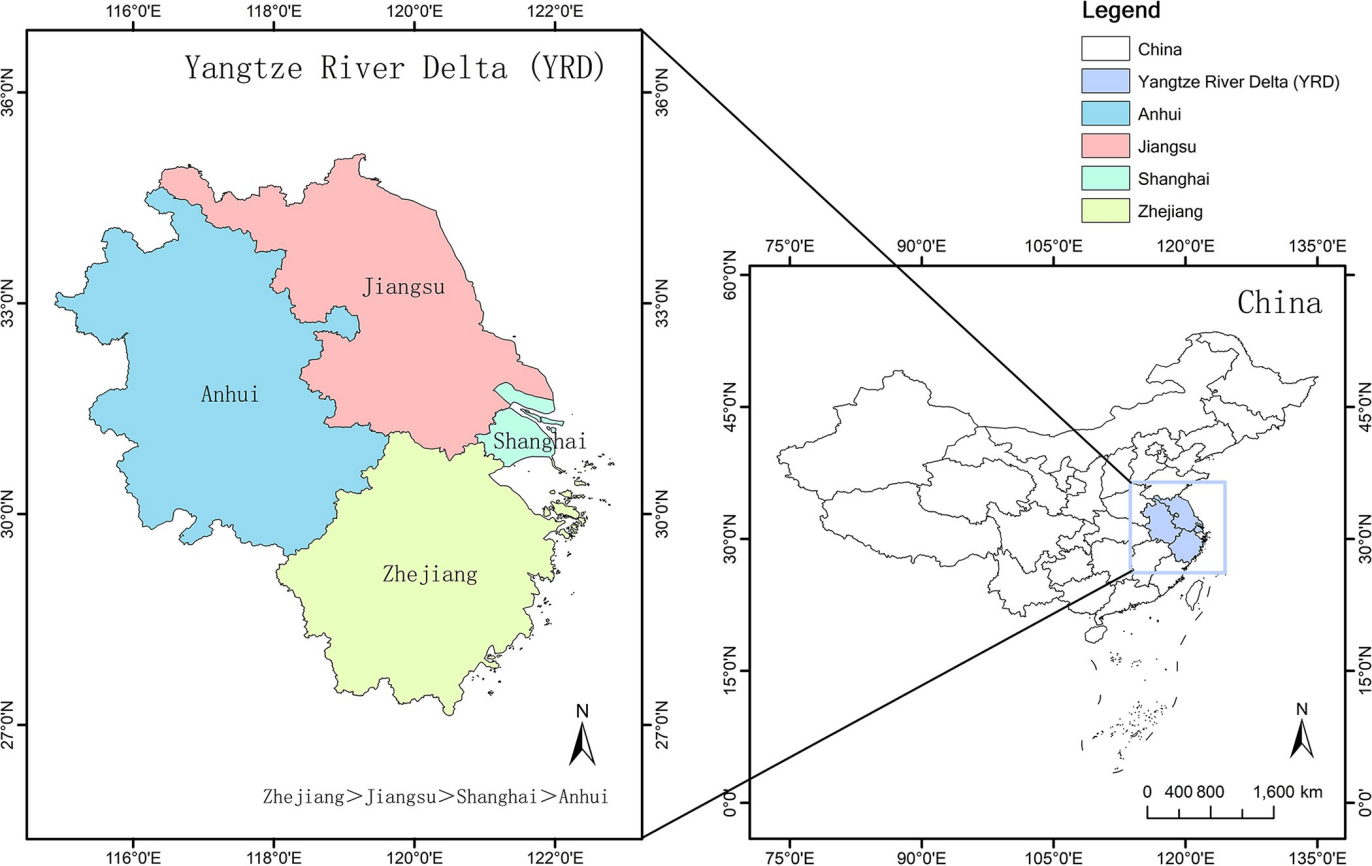
Geographic location map of the YRD region. Source: The primitive base map from the website of the Ministry of Natural Resources of China (http://bzdt.ch.mnr.gov.cn/) was plotted by ArcGIS 10.8 software, and its Drawing Review Number is GS (2023) 2763 and GS (2020) 3189.

China’s eastern shoreline is home to the YRD area. Among the most open and developed areas of the country, this region comprises the provinces of Jiangsu, Zhejiang, Anhui, and Shanghai Municipality. It is situated in the center of China’s coastal economy. Its singular and peculiar geographic situation situates it adjacent to the Qiantang River, which borders the province of Fujian, in the south, the YRD in the north, Chongming Island in the west, and the East China Sea in the east. The region possesses rich resources and labour advantages, attracting a large number of industries and enterprises to gather and form a perfect industrial chain and supply chain. Secondly, the YRD region is conveniently located with advanced land, sea and air transport networks, and is an important node for global economic exchanges. It also boasts numerous tertiary institutions, research institutes and renowned science and technology enterprises. All things considered, the concentration of these resources offers strong support for fostering the excellent growth of the digital creative industries (Fig 1).

### Research methodology

To comprehensively grasp the determinants behind the incredible progression of digital innovative industries in the YRD vicinity, this observe employs the Entropy Weight-TOPSIS technique for facts evaluation. The Entropy Weight technique, primarily employed for multi-criteria decision analysis, serves to ascertain the weights of numerous indicators. Weights are established through the utilization of information entropy derived from the data, with the entropy value’s magnitude serving as an indicator’s representation of significance within the entirety [28]. The strength of the entropy weight method lies in its ability to mitigate the impact of subjective elements on weight allocation, offering a comparatively objective approach to weight determination. It is widely used in multi-criteria decision-making problems, especially in cases involving a large number of indicators and more complex data, entropy weight method effectively solves the problem of indicator weight allocation. The TOPSIS method is often used as a method for multi-criteria decision analysis, aiming to help make optimal decisions. It proves suitable for situations where decision-making involves numerous indicators and options, allowing for a comprehensive assessment of indicator weights and program performance. This, in turn, furnishes decision-makers with a scientifically grounded basis for making informed choices. The essential thought of the TOPSIS method involves contrasting every application against both the nice ideal solution and the negative perfect answer. Subsequently, comprehensive evaluation indicators are established by assessing the likeness or closeness of each program to these two ideal solutions. The positive ideal solution refers to the program that attains the highest value across all indicators, whereas the negative ideal solution pertains to the program that attains the lowest value across all indicators [29, 30].

First, the entropy weight method is used to determine indicator weights. Next, the TOPSIS method is applied. This process includes normalization steps, distance calculations between solutions, and ideal/negative ideal solutions. Finally, a thorough evaluation index is derived.

Diverging from the traditional TOPSIS method, the entropy weight-TOPSIS approach integrates the weights received thru the entropy weight technique with the index information at some point of the computation of the complete assessment index. Within the context of a thorough assessment, this integration best utilizes the burden insights produced by the entropy weight technique.

The entropy-weight-TOPSIS method was selected due to its ability to objectively assess the influence of multiple factors on the development of digital creative industries. By integrating both innovation-driven and policy-driven factors, this method provides a more comprehensive understanding of the synergies between these variables, addressing the limitations of prior studies that focus solely on single-factor analysis. This approach allows for a holistic evaluation of the dynamic interactions that drive regional industrial growth.

The entropy weight method’s use leverages its intrinsic objectivity in weight determination, enabling a more objective and thorough assessment of the combined impact of many variables on the development of the digital creative industries [31, 32]. This enhances the scientific validity and dependability of the comprehensive evaluation outcomes, diminishing subjective biases, and furnishing decision-makers with more valuable information and guidance. Furthermore, it aids in uncovering pivotal driving forces behind the high-quality progression of digital creative industries.

### Sources of data

For this study, some publicly available data is collected in an effort to better understand the factors driving the YRD region’s high-quality development in the digital creative sectors. This article solely gathers statistics for each province and city for the years 2017–2022, taking into account data availability. The statistical yearbook of China’s cultural and allied sectors, many province and city statistical yearbooks, and other pertinent industrial statistics databases provided the pertinent data that were used. In studying the geographical distribution of digital creative industries in the YRD region, mapping tools, such as mapping software or GIS (Geographic Information System), are used. Through these geographical distribution maps, we can visualise the regional data distribution characteristics and agglomeration of digital creative industries in the YRD region.

### Building the indication system

The study makes evident the wide range of factors influencing the growth of the digital creative industries. 24 assessment indicators are selected by this study from a pool of relevant characteristics, including talent reserves, market operation, technical innovation, regulatory support, and regional resources. It also refers to some scholars’ suggestions regarding the development planning of digital creative industries. The statistical data related to digital creative industries is combined in this study. Constructing a three-level indicator system including target layer, quasi-measurement layer and programme layer (Table 1). Talent, policy, market, innovation, and technology are the main drivers of high-quality growth in the YRD region’s digital creative industries. These factors also propel the quasi-measurement layer.

**Table 1:** The YRD region’s digital creative industries have high-quality development, according to a system of factors.

desired level	quasi-testing stratum	at the program level	unit of measurement	Weights (entropy computation results for 2022 weights)	bibliography
**A1 An analysis of the driving forces behind the YRD region’s superior growth in the digital creative industries**	**B1 Innovative Technology Driven**	C1 number of granted patent applications (+)	pieces	0.0573	[5, 11, 33]
		C2 Total number of registered copyright agreements (+)	copies	0.0306	
		C3 Research and experimental development (R&D) funding (+)	million	0.0391	
		C4 Number of enterprises with innovation strategy objectives (+)	units	0.0521	
		C5 Number of voluntary registrations of works (+)	pieces	0.0294	
	**B2 Policy Driver**	C6 Degree of impact of policy support for enterprise innovation and development (+)	%	0.0492	[34]
		C7 Financial expenditure on education (+)	billion	0.0428	
		C8 Financial expenditure on science and technology (+)	billion	0.0345	
		C9 Financial expenditure on culture, sports and media (+)	billion	0.0338	
	**B3 Market Driven**	C10 Number of strategic emerging industry project transactions (+)	items	0.0483	[3, 10]
		C11 Per capita consumption expenditure on culture and recreation (+)	Yuan	0.0317	
		C12 Gross regional product (+)	billion	0.072	
		C13 Total income of radio and television business units (+)	million	0.0529	
		C14 Total assets of enterprises in cultural and related industries (+)	million	0.0409	
		C15 Frequency of public television programmes broadcast throughout the year (+)	hours	0.0282	
		C16 Frequency of public broadcasting programmes throughout the year (+)	hours	0.0284	
	**B4 Talent Driven**	C17 Number of R&D topics in higher education (+)	units	0.035	[8, 35]
		C18 Number of students graduating (completing) from general schools (+)	persons	0.0335	
		C19 The number of employees engaged in experimental development and research (R&D) (+)	persons	0.0681	
		C20 The quantity of workers in mass culture organizations (+)	persons	0.0396	
	**B5 regional drive**	C21 Mobile Internet user penetration (+)	10,000 households	0.0363	[36]
		C22 Number of public libraries (+)	units	0.0288	
		C23 Number of cable radio and television subscribers	Households	0.0512	
		C24 Area of land for public facilities (+)	Km^2^	0.0364	

Cutting-edge, technologically driven marker B1, which also includes the quantity of authorized patent applications (C1); the quantity of registered copyright agreements (C2); money for experimental development (R&D) and research (C3); the quantity of businesses that have developed creative strategic goals (C4); and the number of works voluntarily registered (C5). All of these factors indicate that the digital creative sector should concentrate on technical and creative industry research, either directly or indirectly; the greater the indicator’s value, the better the standard of the sector’s industrial development.

The policy-driven marker is B2, including the degree of influence of policy support for enterprise innovation and development (C6); financial expenditure on education (C7); financial expenditure on science and technology (C8); and financial expenditure on culture, sports and media (C9). The state now places a high value on the digital creative sector, and the degree to which policies influence enterprise innovation and development suggests that the business sector needs a lot of policy support to establish new enterprises related to the incubation of the digital creative sector [37]. Furthermore, financing for the arts, sciences, sports, media, and education can help to sustain the rapid expansion of the digital creative industry.

The number of strategic emerging industry project transactions (C10), per capita expenditure on culture and recreation (C11), Gross Regional Product (C12), total revenue of broadcasting and television business units (C13), total assets of cultural and related industry enterprises (C14), annual public radio program broadcasting hours (C16), and total revenue of broadcasting and television business units (C13) all contribute to the market-driven mark, which is B3. The strength of the market determines how much of an influence it has on the growth and development of the digital creative industries; the stronger the market is in terms of project transactions, consumer spending, and corporate profits [38].

Talent Driver marked as B4 included: the number of R&D themes in higher education (C17), the number of regular school graduates (C18), the number of employees of mass cultural organizations (C20) and the quantity of individuals working on experimental development and research (R&D) (C19). The quality of the talent pool required to sustain the further growth of the digital creative sector is directly impacted by the proportion of general school graduates among them. The relevance of talent resources in fostering the growth of digital creative industries is demonstrated by the quantity of R&D topics given in higher education, the quantity of R&D specialists, and the quantity of practitioners in mass cultural organizations [39].

The factors that comprise the regional driver, denoted by the letter B5, are the quantity of public libraries (C22), the quantity of cable radio and television subscribers (C23), the percentage of people using mobile devices to access the Internet (C21) and the amount of land designated for public spaces (C24). The level of regional urban construction can be measured by looking for these indicators; the higher the level, the larger the chance to grow digital creative industries.

### Data calculation process

#### Eight of indicators is determined using the entropy weight approach

Make a preliminary assessment matrix. Use the symbol X_ij_ to represent the numerical value of the i city for the j evaluation indicator, given that a region has m cities and n indicators of growth drivers. such that j∈[1,n] and i∈[1,m]. We are able to acquire the starting matrix X, as seen below:

$$X=\left[\begin{array}{cccccc} X_{11} & X_{12} & \cdots & X_{1i} & \cdots & X_{1n}\\ X_{21} & X_{22} & \cdots & X_{2j} & \cdots & X_{2n}\\ \vdots & \vdots & \ddots & \vdots & \ddots & \vdots \\ X_{i1} & X_{i2} & \cdots & X_{ij} & \cdots & X_{in}\\ \vdots & \vdots & \ddots & \vdots & \ddots & \vdots \\ X_{m1} & X_{m2} & \cdots & X_{mj} & \cdots & X_{mn} \end{array}\right]$$ (1)

First, ascertain the weight P_ij_ of the j evaluation indication in the i area before computing the entropy weights of the indicators:

$$P_{ij}=y_{ij}/\sum_{i=1}^{m}y_{ij}$$ (2)

Also, let e_j_ denote the entropy value of the j evaluation index:

$$e_{j}=\frac{1}{\ln\;m}\sum_{i=1}^{m}P_{ij}\;\ln\;P_{ij}$$ (3)

Using the information provided, calculate the entropy weight W_j_ for each index in the Impact Indicator System of Driving Factors for High-Quality Development of Digital Creative Industry.


$$W_{j}=\frac{1-e_{j}}{n-\sum_{j=1}^{n}e_{j}}$$ (4)

#### The YRD region’s digital creative industries are evaluated for overall high-quality development using the TOPSIS approach

A weighted normalization matrix Y is calculated by weighting the normalisation matrix Z.


$${Z=(z_{ij})}_{m\times n}={\left(w_{j}\times y_{ij}\right)}_{m\times n}=\left[\begin{array}{cccc} z_{11} & z_{12} & \cdots & z_{1n}\\ z_{21} & z_{22} & \cdots & z_{2n}\\ \vdots & \vdots & & \vdots \\ z_{m1} & z_{m2} & \cdots & z_{mn} \end{array}\right]$$ (5)

By identifying both benefits and drawbacks ideal solutions and deriving the associated positive and negative ideal solution vectors, U^+^ and U^−^, one may determine the highest and worst values of the effect of the drivers of the digital creative sector:

$$Positive\;Ideal\;Solution:U^{+}=\left\{(\underset{1\leq i\leq m}{\max}Z_{ij}|j\in J^{+}),(\underset{1\leq i\leq m}{\min}Z_{ij}|j\in J^{-})\right\}=\{z_{1}^{+},z_{2}^{+},\cdots ,z_{n}^{+}\}$$

$$Negative\;Ideal\;Solution:U^{-}=\left\{(\underset{1\leq i\leq m}{m\mathrm{in}}Z_{ij}|j\in J^{+}),(\underset{1\leq i\leq m}{\max}Z_{ij}|j\in J^{-})\right\}=\{z_{1}^{-},z_{2}^{-},\cdots ,z_{n}^{-}\}$$ (6)

where the sets of positive and negative indications are represented, respectively, by J^+^ and J^−^.

Using the Euclidean metric calculation approach, find the distances $D_{ki}^{+}$ and $D_{ki}^{-}$, respectively, between the indicator evaluation vector and the positive ideal solution U^+^ and negative ideal solution U^−^ of the YRD area.


$$D_{ki}^{+}=\sqrt{\sum_{j=1}^{n}{\left(z_{ij}-z_{j}^{+}\right)}^{2}},i\in [1,m]$$

$$D_{ki}^{-}=\sqrt{\sum_{j=1}^{n}{\left(z_{ij}-z_{j}^{-}\right)}^{2}},i\in [1,m]$$ (7)

Assessment, in a high-quality manner, of the relative proximity of the elements impacting the growth of the digital creative industries. Let C_I_ represent the estimated distance between i city and the complete, high-quality expansion of the digital creative industries.


$$C_{i}=\frac{D_{i}^{-}}{D_{i}^{+}-D_{i}^{-}},i\in [1,m]$$ (8)

A higher C_i_ number denotes a higher degree of comprehensive, high-quality growth in i city’s digital creative industry; a lower value denotes the reverse.

## Results

### A thorough evaluation of the YRD region was carried out between 2017 and 2022

Between 2017 and 2022, the digital creative industries’ overall degree of development in the YRD region increased from 0.264 to 0.709 (designated H1, H2, H3, H4, H5, and H6, respectively). By 2022, the YRD area’s digital creative industries, with ratings of 0.264, 0.281, 0.351, 0.458, 0.707, and 0.709, will be completely developed and of the highest grade (Table 2). This illustrates even further how China has been actively pushing the framework of its continuous support for the development of strategically significant new sectors in recent years, with the main industry being the rapidly growing digital creative sector. Digital creativity is growing at a quick pace, with the YRD area at its center, at the center of China’s coastal economy. Now that it has fully recovered from the worst impacts of the recent crown pandemic, this company is growing rapidly.

**Table 2:** Closeness to superior development of digital creative industries in the YRD area, 2017–2022.

TOPSIS evaluation calculations				
Items	optimum solution distance that is positive $D_{ki}^{+}$	negative distance for the optimum solution $D_{ki}^{-}$	relative closeness C_I_	arranging the outcomes
H1	0.202	0.072	0.264	6
H2	0.181	0.071	0.281	5
H3	0.166	0.090	0.351	4
H4	0.140	0.119	0.458	3
H5	0.069	0.166	0.707	2
H6	0.080	0.195	0.709	1

The YRD region’s digital creative industries did not show any appreciable progress in terms of overall quality of development between 2017 and 2018. The indicator with the largest weight share (0.0971) is the number of ordinary undergraduate and specialist graduates (C18) from general schools; the weight coefficients of the other indicators range from 0.020–0.066. The YRD region had a 16,497 increase in the number of ordinary undergraduate and specialist graduates (completion) from ordinary schools throughout the course of the year, from 1,223,095 to 1,239,592. The digital creative industry’s general standard of high-quality development was enhanced by this expansion. Four indicators were raised in order to assist the superior growth of the digital creative industry: total income of broadcasting and television enterprise units (C13); expenditure on research and experimental development (R&D) (C3); number of works voluntarily registered (C5); number of project transactions in strategic emerging industries (C10); and number of pieces of project income (55,579, 356, and 761,764,000 yuan, respectively). The per capita expenditure on consumption of culture and entertainment (C11) dropped from RMB 6,142.4 to RMB 5,698, which is a positive indicator. This fall had an impact on the annual growth rate of the comprehensive level. This is related to the global economic imbalance in 2017, where the overly rapid pursuit of development speed and maximisation of benefits led to inflation caused by overconsumption, and the people’s happy living standards could not be met, thus leading to a decrease in per capita cultural and recreational consumption expenditure. The digital economy was mentioned as a keyword to start a long-term study of its development strategy approach in the 2017 government work report and the 19th Party Congress report. The substantial rise of the digital creative sector in the following years was largely due to this attempt.

The YRD region’s digital creative industries had a modest increase in overall high-quality growth between 2018 and 2019. Metrics that indicate positive growth include the number of project transactions in strategic emerging industries (C10), the per capita expenditure of residents on culture and entertainment (C11), the number of employees in mass cultural institutions (C20), the quantity of individuals working on experimental development and research (R&D) (C19), and the quantity of employees in R&D (C11). These indicators show increases of 3,955 items, 292.8 yuan, 241,823 people, and 253 people. In terms of negative growth indicators, there was a decrease of 14,381, 75,493,410,000 yuan, and 893 items in the number of R&D funding (C3), voluntarily registered works (C5), cable broadcasting and television subscribers (C23), ordinary undergraduate and specialist graduates (C18), and hours of public television programs aired year-round (C15). The occurrence of a slight increase in the composite level is caused by the weight coefficients of the positive growth indicators being generally bigger than those of the negative growth indicators. The YRD region’s digital creative business, however, fared poorly overall in 2018, despite the country’s spectacular expansion. This was caused by factors such as a fall in general school graduates, a reduction in funding for research and development, and a decline in records such as artists’ works.

The YRD region’s digital creative industries expanded at a thorough and superior rate in 2019–2020. The number of copyright contract registrations (C2), revenue spent on science and technology (C8), per capita resident spending on culture and entertainment (C11), annual public TV program broadcasting hours (C15), and 260, 2.289 billion yuan, 2,519.4 yuan, and 16,422 hours, respectively, are some indicators that show a decline. The remaining metrics demonstrated expansion. A number of measures of innovation technology and policy-driven aspects have increased over time. These include the quantity of approved patent applications (C1), the amount of money spent on R&D (C3), the number of companies that have set goals for their innovation strategies (C4), the quantity of works that have voluntarily registered (C5), and the degree to which business innovation and development has been impacted by policy support (C6). The technology dividend country is still developing, the digital creative sector is expected to grow in 2019 under favorable conditions, and the industry’s industrial chain is continuing to grow and integrate various enterprises. The YRD area’s digital creative sector is growing because of this environment.

In the YRD area, there was a discernible increase in the overall degree of high-quality development in the digital creative industries between 2020 and 2021. The number of ordinary undergraduate and specialist graduates of ordinary schools (C18), the total income of broadcasting and television enterprise units (C13), the amount spent on experimental development and research (R&D) (C3), the quantity of works that are willingly registered (C5), the per capita expenditure of residents on culture and entertainment (C11), and the number of transactions of the projects of the strategic emerging industries (C10) are the indicators with a high proportion that all show an increase in terms of the weight coefficients of the indicators. There has been an increase of 293,347 individuals, 583,202,700 yuan, 105,744,525,000 yuan, 220,845 pieces, 573.3 yuan, and 5,038 things in each of the four indices. This is because the YRD region actively addressed the epidemic’s effects in 2020 and encouraged the growth of neighborhoods centered around digital technology. Mobile gaming, online casting, digital reading, and other modules have all experienced exponential growth in the network cultural industry segment. The local digital creative sector has had a notable upturn in development as a result of this growth.

In general, the YRD area’s high-quality development in the digital creative industries appears to have improved in 2021-2022. In terms of indicators, many positive indicators show reverse growth, such as the number of patent applications authorised (C1), the number of copyright contracts registered (C2), the degree of impact of policy support for innovation and development of enterprises (C6), the number of transactions of projects in strategic emerging industries (C10), the per capita consumer spending on culture and entertainment (C11), the total assets of enterprises in the cultural and related industries (C14), the number of public libraries (C22) Number of cable radio and television subscribers (C23), and area of land for public facilities (C24). The excellent development of digital creative businesses in the YRD region is not greatly impacted by the low weighting factors of these variables. In 2021, however, the digital economy started to quicken the pace of the broader economic and social development in one city and three provinces in the YRD region-where both technological innovation and the growth of digital infrastructure had developed consistently. Consequently, it has compelled the digital creative sector to get ready for a major innovation.

### Three provinces and one city in the YRD region are contrasted

#### Comparing six years’ worth of data together

**Fig 2:**
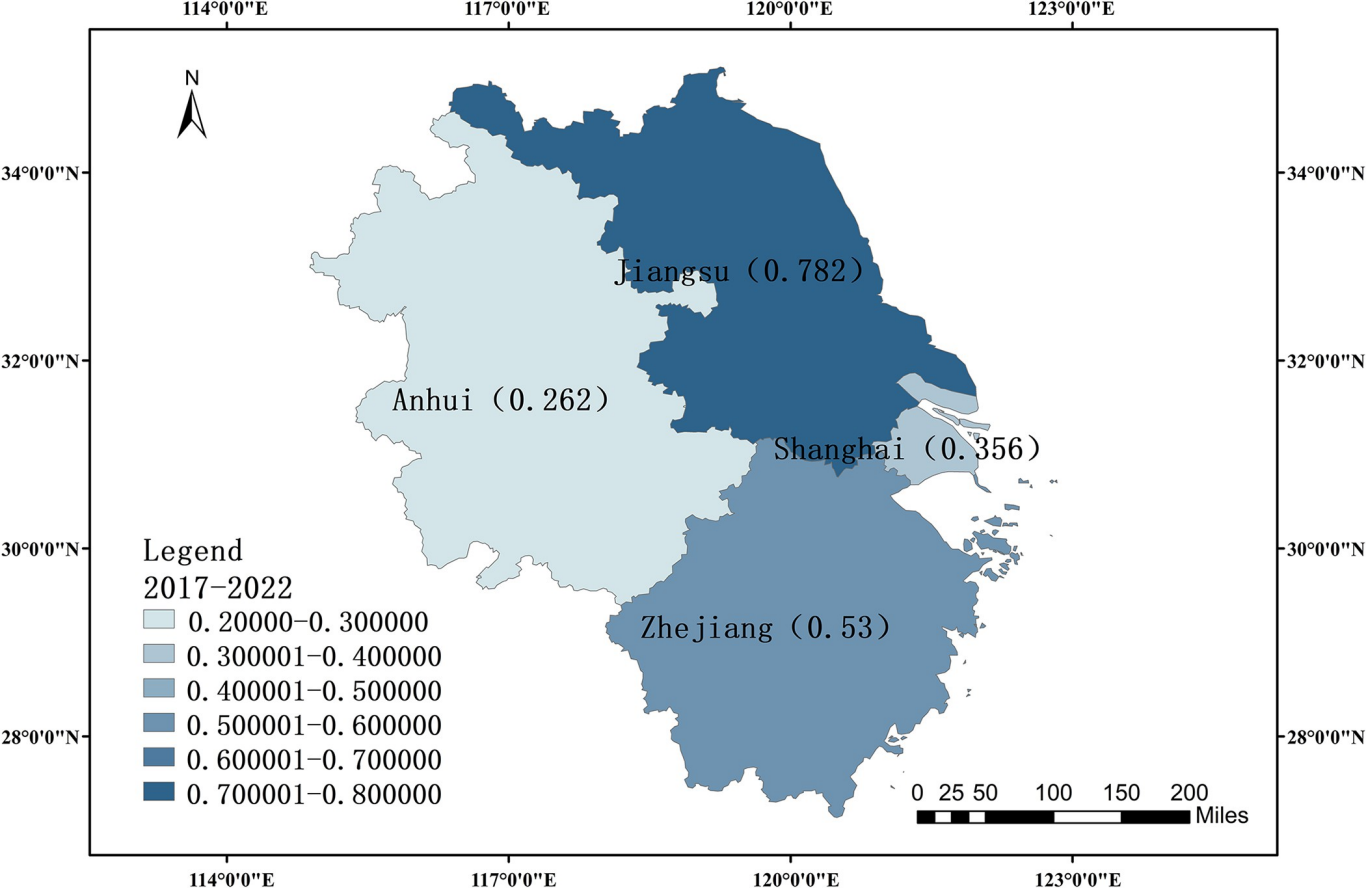
Comprehensive evaluation level of the YRD region over a six-year period. Source: The primitive base map from the website of the Ministry of Natural Resources of China (http://bzdt.ch.mnr.gov.cn/) was plotted by ArcGIS 10.8 software, and its Drawing Review Number is GS (2020) 3189.

In one city and three provinces in the YRD area, this study used the entropy weight TOPSIS technique to evaluate the overall relative closeness of high-quality growth of digital creative industries during a six-year period, from 2017 to 2022. To better understand the spatial distribution patterns of the high quality growth of digital creative industries in the YRD area, the relative proximity of these sectors is divided into six intervals using ArcMap 10.8. Next, a map of each region’s comprehensive evaluation level during a six-year period is created (Fig 2).

With a relative proximity of 0.782 in Jiangsu province, Jiangsu has the best comprehensive degree of development for the digital creative sector throughout the 2017–2022 period, far greater than the other three areas. Zhejiang Province ranks second at 0.53 with a medium rate of development in the region’s digital creative industry. With relative proximity of 0.356 and 0.262, respectively, Shanghai and Anhui Province are relatively impoverished, and the digital creative sector is not at a good stage of growth. The economic standing of each area should be taken into consideration. Anhui Province and Shanghai Municipality have extremely low GDPs in the YRD region when compared to the other two provinces and municipalities, which exhibit a cliff-like separation. Jiangsu Province has the highest GDP, which affects the larger level of industrial development due to the extraordinary leading economic level.

#### Six-year comparison, year-by-year

**Fig 3:**
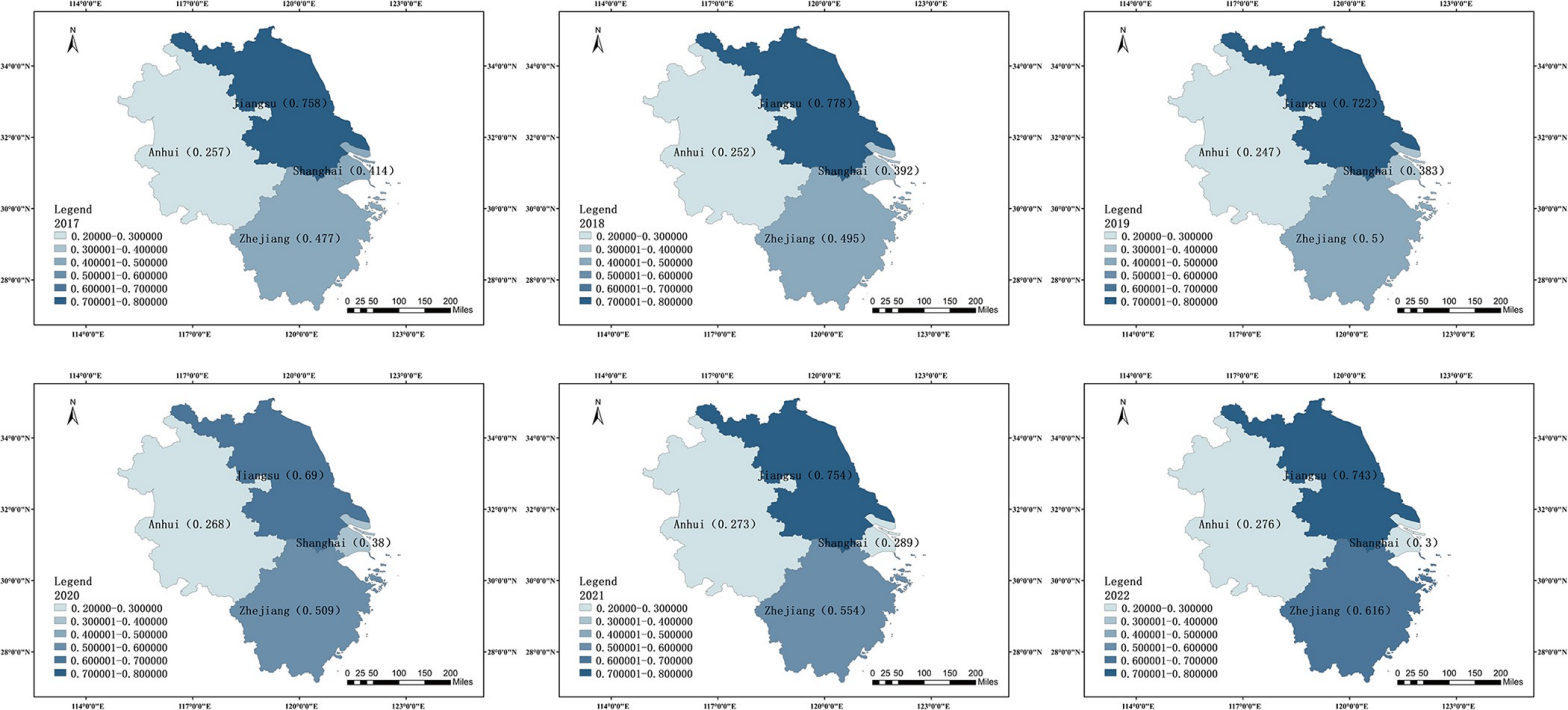
Year-by-year composite level of the YRD region, 2017–2022. Source: The primitive base map from the website of the Ministry of Natural Resources of China (http://bzdt.ch.mnr.gov.cn/) was plotted by ArcGIS 10.8 software, and its Drawing Review Number is GS (2020) 3189.

In one city and three provinces in the YRD area, the relative closeness of the development level of digital creative industries was computed successively from 2017 to 2022 using the entropy weight TOPSIS technique. With ArcMap 10.8, the distribution map of each area’s evaluation level in the same year was made possible, and the relative proximity of the comprehensive level of the development of the digital creative sector year over year was divided into six different intervals (Fig 3).

The relative proximity of Shanghai in 2017–2022 is 0.414, 0.392, 0.383, 0.380, 0.289, 0.300, showing a regular downward trend. 2017 The digital creative industry is developing at the highest rate in Shanghai, however this is declining yearly, in part due to the size of the surrounding region. Over time, the idea of "one main and multiple vice" has evolved to accommodate the YRD continuous expansion in the digital creative economy, with Jiangsu, Zhejiang, Anhui, and other cities as the vice and Shanghai as the center. As a result, Shanghai’s comprehensive level progressively starts to fall as the years pass.

The relative proximity of Jiangsu Province in 2017–2022 is 0.758, 0.778, 0.722, 0.690, 0.754, 0.743,and the whole shows a rising state. In comparison to the preceding five years, Jiangsu Province’s overall level of development in 2020 is comparatively low. This can be attributed to the province’s lack of policies pertaining to the digital creative industry, the low level of innovation exhibited by its enterprises, and the low volume of project transactions. The number is small, and the strength of policy support is not strong enough. 2020 is impacted by the epidemic more so than the other years, which has an impact on the excellent growth of the digital creative business.

Zhejiang Province’s relative proximity is 0.477, 0.495, 0.500, 0.509, 0.554, and 0.616 from 2017 to 2022, respectively, and it is steadily increasing overall. The relative closeness to Zhejiang Province peaks in 2022, suggesting that the digital creative sector in the area is steadily growing. The province of Zhejiang is dedicated to supporting the expansion of the digital creative sector. Renowned for its creative approaches to urban planning, the province was the first to offer 10 measures of assistance and a three-year action plan to help foster the growth of the digital economy. Zhejiang Province is simultaneously building its digital creative industry in line with the demands of a healthy industrial chain, the implementation of an innovation chain, the refinement of the capital chain, the organization of the talent chain, the expansion of the service chain, and the optimization of the ecological chain. In such a setting, Zhejiang Province’s digital creative industries have been developing at a higher and higher level.

Anhui Province’s relative proximity from 2017 to 2022 is 0.257, 0.252, 0.247, 0.268, 0.273, and 0.276, respectively, indicating a marginal overall rise. Over the last six years, Anhui Province’s relative proximity has essentially remained constant, but slightly increasing annually. Anhui Province is aggressively promoting eleven emerging sectors, including the digital creative sector. The province government, through the Anhui Party Committee, established a provincial digital creative industry promotion group work task force to further the industry’s development. Documents from the Anhui Province Culture and Tourism Department in recent years show this. These initiatives are the main driver of the improvement in the caliber of development of the Anhui Province’s digital entrepreneurship sector.

## Discussion

By analyzing the key drivers in the YRD region between 2017 and 2022, this study reveals the important roles of technological innovation, policy support, market forces and talent resources in promoting the high-quality development of digital creative industries. These findings not only answer the question of the differences between provinces and cities in the region in the development of the industry, but also provide a new empirical basis for the theoretical study of the digital creative industry, especially the exploration of the synergistic effect of multi-factor driving force, which provides a new perspective for the academic community. This study complements the existing literature on the synergistic effects of policy and innovation environments, such as Hosseini, E et al.’s view that technological innovation and policy support are the core driving forces for the development of creative industries, further expanding the academic discussion in this area [12, 36].

In a regional comparison, Jiangsu and Zhejiang perform particularly well, thanks to their governments’ continued investment and policy support for innovation and talent. For example, Jiangsu Province significantly increased its financial investment in science and technology innovation during the 14th Five-Year Plan period, while Zhejiang Province introduced a large number of high-end technical talents through the strategy of “Strengthening the Province with Talents”. These initiatives have effectively promoted the vigorous development of digital creative industries in both places [23]. In contrast, although Shanghai was a leader in the early stage of the digital economy, it has gradually lost its strength in talent and technology export due to geographic and market constraints. Anhui, as a late-stage region, has realized a faster rise through policy guidance and government support, showing the potential for regional development and late-stage advantages. Similar patterns of combining government support and market strategies have also appeared in other countries, such as the creative industries in South Korea and Japan, which are similar to the case of the YRD [26].

The findings also suggest that the experience of the YRD has strong general applicability. Other similar economic regions, such as Beijing-Tianjin-Hebei and the Pearl River Delta, also have policy and market-driven creative industry development [40]. Niu Junjun’s study points out that global and local markets have different mechanisms for influencing creative industries, but policy support and market expansion remain key drivers [23]. In addition, the Seoul region in South Korea relies on large-scale government R&D investments and policy incentives to form a high degree of integration of innovation and cultural creativity, a model that is important for the development of creative industries in other regions [12]. Future research could further validate the generalizability of these drivers and explore the long-term impact of emerging technologies (e.g., blockchain and artificial intelligence) on digital creative industries.

Based on the above findings, this study proposes the following countermeasures: first, regions should further strengthen their support for innovation drive and talent introduction at the policy level. Jiangsu and Zhejiang can continue to increase support for high-end talents and technology enterprises, while Shanghai needs to focus on optimizing the market environment and improving its own technological innovation capacity. Anhui should consolidate its existing policy advantages and strengthen the integration of industrial and innovation chains. These measures will help enhance the overall competitiveness of the region and realize the synergistic development of digital creative industries.

Through an examination of the key drivers of the digital creative industries in the YRD area, this report provides detailed insights into the unique processes of each driver in various locations and suggests appropriate policy measures. These discussions are not only of guiding significance for the digital creative industries in the YRD region, but also provide references for other regions, helping them to maintain the sustained competitiveness of their creative industries against the backdrop of the ever-changing global market environment. Future research could be further expanded to verify the generalizability of these findings and explore how emerging technologies affect the long-term development of digital creative industries.

## Conclusion

After conducting a comprehensive examination of the superior growth of digital creative industries in the YRD area between 2017 and 2022, this research selects 24 indices from five level systems: policy support, market operation, talent reserve, technical innovation, and regional resources. Then, using these indexes, an index system of driving forces for the superior growth of the digital creative industries is created. It quantitatively assesses the overall level of development of the digital creative industries in the region using the entropy-weight-TOPSIS technique. The dual route model of innovation-driven and policy-driven was presented after a quantitative examination was completed. This theoretical framework establishes a strong basis for future study while also offering a fresh viewpoint on the evolution of the digital creative industries.

The study’s findings indicate that there is a strong development momentum in the digital creative business in the YRD area, with a notable increase trend observed between 2017 and 2022. In particular, the provinces of Jiangsu and Zhejiang are at the forefront of the growth of the digital creative industries. They have a number of advantages over other regions, including the ability to innovate in science and technology, the ability to build digital infrastructure, and policy support, all of which contribute significantly to the industry’s rapid development. Shanghai lags behind Jiangsu and Zhejiang in terms of innovation drive and regulatory support for digital creative sectors, despite its advantages in terms of economic aggregate and market scale. The digital creative industry in Anhui Province started relatively late, but under the policy promotion, it has also shown a fast development speed in recent years.

Technological research and development, creative design, and digital transformation are key aspects where innovation-driven growth is evident. These elements directly affect the competitiveness and market acceptance of digital creative products. On the other hand, a policy-driven environment reflects the external frameworks that the government has established to support the growth of digital creative industries, including the encouragement of digital infrastructure development, promotion of industrial agglomeration, and favorable regulatory implementations. The high-quality growth of digital creative industries in the YRD region is mutually promoted by these interrelated aspects.

In light of these findings, several key policy recommendations emerge to further promote the development of digital creative industries across the YRD region.

Strengthening Innovation and Talent Support in Jiangsu and Zhejiang: Both provinces should continue to invest in high-end talents and technological enterprises. By expanding financial support for research and development (R&D), providing innovation incentives, and fostering collaboration between universities, research institutes, and industries, they can further boost industry growth.

Optimizing the Market Environment in Shanghai: Although Shanghai was once a leader in the digital economy, it now faces challenges in maintaining its competitive edge. Policy efforts should focus on optimizing the market environment, enhancing technological innovation, and improving talent retention mechanisms to revive its digital creative sector.

Leveraging Policy Advantages in Anhui: Anhui Province has made significant progress through policy-driven initiatives. It should further consolidate its existing policy advantages and strengthen the integration of industrial and innovation chains. Supporting digital startups and fostering creative industry clusters will solidify Anhui’s position as an emerging force in the digital creative industry.

## Supporting information

S1 Data Evaluation and analysis of driving factors for high quality development of overall digital creative industry in YRD, 2017–2022 raw data. (XLSX)

S2 Data Evaluation and analysis of the level of high-quality development of digital creative industries in three provinces and one city within the YRD region, 2017–2022 raw data. (XLSX)

S3 Data Year-by-year comparison of the evaluation and analysis of the level of high-quality development of digital creative industries in the YRD region—2017 raw data. (XLSX)

S4 Data Year-by-year comparison of the evaluation and analysis of the level of high-quality development of digital creative industries in the YRD region—2018 raw data. (XLSX)

S5 Data Year-by-year comparison of the evaluation and analysis of the level of high-quality development of digital creative industries in the YRD region—2019 raw data. (XLSX)

S6 Data Year-by-year comparison of the evaluation and analysis of the level of high-quality development of digital creative industries in the YRD region—2020 raw data. (XLSX)

S7 Data Year-by-year comparison of the evaluation and analysis of the level of high-quality development of digital creative industries in the YRD region—2021 raw data. (XLSX)

S8 Data Year-by-year comparison of the evaluation and analysis of the level of high-quality development of digital creative industries in the YRD region—2022 raw data. (XLSX)
